# Does internationalization improve environmental disclosure willingness and quality? The moderating role of green investors

**DOI:** 10.1371/journal.pone.0307638

**Published:** 2024-09-11

**Authors:** Juan He, Shuang Yang

**Affiliations:** 1 School of International Business, Southwestern University of Finance and Economics, Chengdu, Sichuan, China; 2 Financial Department, Qingdao Haijian Investment CO., Ltd., Qingdao, Shandong, China; University of Madeira / NOVA Lincs, PORTUGAL

## Abstract

Environmental issues have gradually become a key concern for society. The public has been paying increasing attention to corporate environmental disclosure and performance. With the “go global” trend, more and more enterprises are looking to overseas markets for new technologies and resources. Multinational enterprises (MNEs) are facing more challenges than domestic enterprises. To remain competitive and sustainable, enterprises from developing countries need to gain a foothold in developed countries. We explore how MNEs’ internationalization impacts environmental disclosure, specifically focusing on the role of green investors as stakeholders. We draw evidence from Chinese-listed MNEs, with a total of 4,709 panel data observations. For the main analysis, we use a fixed effect model. The findings suggest that a higher level of internationalization can improve both the willingness and quality of environmental disclosure for MNEs, and this relationship is further strengthened by green investors. A heterogeneity analysis reveals that the positive effect of internationalization on environmental disclosure is mainly present in state-owned enterprises (SOEs) and developed host countries. We find that external pressure from host countries motivates MNEs to increase environmental disclosure willingness and quality. This study provides valuable insights for MNEs from emerging economies on how to achieve legitimacy and a positive reputation in overseas markets through environmental disclosure strategies. This study proposes the importance of green investors on environmental disclosure issues from a stakeholder perspective and provides new theoretical insights for environmental policy reform in developing countries such as China.

## 1. Introduction

The following six Sustainable Development Goals (SDGs) set by the United Nations are closely related to the environment: clean water and sanitation, clean energy, sustainable consumption and production, climate action, sustainable development of ocean resources, and sustainable ecosystem development. As environmental issues are global, enterprises are the main actors in achieving these goals. The public has been paying increasing attention to corporate environmental disclosure and performance; enterprises are being pressured to disclose more information about the environmental impacts of their products and production [1]. In recent decades, MNEs have faced more challenges than domestic enterprises. With their liability of origin, MNEs face disadvantages in the host country because they operate outside their institutional context [2, 3]. Therefore, for long-term survival and development, MNEs should consider gaining legitimacy in foreign contexts [4]. Information disclosure enlightens host-country stakeholders about an enterprise’s products or services. Traditionally, financial information is the most essential information for an enterprise because it allows shareholders to determine whether profits are positive or negative. However, sustainable development should also be a key performance indicator. Environmental disclosure is a crucial aspect of sustainable development and a platform for enterprises to demonstrate corporate social responsibility (CSR).

Globalization has increased the number of MNEs, and state-owned MNEs play a significant role in their interests and national strategy. SOEs may be quicker executors of governments’ national industrial policies. To obtain legitimacy from the government, SOEs must contribute to national objectives [5, 6]. Given that the SDGs are global, state-owned MNEs are expected to substantially contribute to environmental disclosure.

Investors are important stakeholders of enterprises and greatly influence corporate management. Non-green investors primarily aim for economic benefits, whereas green investors focus on green and high-quality investment [7, 8]. Consequently, green investment is a combination of financial and environmental objectives [8, 9]. Enterprises with green investors are more likely to promote green action, increase green spending, and improve green governance.

Environmental disclosure embodies CSR. For corporate sustainability, CSR has become increasingly important [10, 11]. Literature on environmental disclosure mainly focuses on domestic market [12–15]. MNEs have become important part of the economy, but studies on environmental disclosure from international perspective are insufficient. Therefore, studying the effect of internationalization on environmental disclosure is crucial, especially in emerging economies, because the institutional systems of emerging markets are weaker than those of developed countries [16]. Additionally, it is more challenging for emerging economies to overcome the liability of origin disadvantages. Investors are important stakeholders of enterprises. Different from traditional investors, green investors focus on green and high-quality investment [7], inevitably exerting significant influence on environmental disclosure of enterprises. However, the literature has rarely examined the effect of green investor as a strategic investor from a stakeholder perspective.

This study contributes to the literature on several important aspects. First, it focuses on the influential factor of environmental disclosure from an international perspective, deepening firm environmental performance research in the domestic market. Second, it proposes refining the environmental disclosure into two aspects, namely, environmental disclosure willingness and quality.Third, given the increasing attention to environmental protection, this study emphasizes the important role of green investors in improving environmental disclosure willingness and quality from a stakeholder perspective.

## 2. Theoretical background and hypotheses development

### 2.1 Environmental disclosure

The literature has examined multiple aspects of environmental disclosure, which can be divided into the effect of environmental disclosure and the factors influencing it.

First, corporate environmental disclosure has multiple effects, such as a significant positive effect on environmental [12] and financial performance [17]. Numerous enterprises implement environmental disclosures as an environmental strategy in response to institutional pressure. Environmental disclosure and innovation improve energy efficiency and provide a competitive advantage in green markets. Enterprises in environmentally sensitive industries can increase their recognition and legitimacy by issuing CSR reports [13] because environmental disclosure is a CSR behavior. Environmental disclosure can also improve satisfaction with environmental governance.

Second, several internal and external factors influence corporate environmental disclosure. Internally, this includes administrative environmental innovations [18], the influence of boards [19, 20], and corporate governance structures [14]. Externally, enterprises have incentives to disclose environmental information to gain legitimacy [13].

### 2.2 Internationalization and environmental disclosure

MNEs face more challenges than domestic enterprises because they must reduce information asymmetry and increase legitimacy and recognition in overseas markets. They also face pressure from both the host and home countries. Operating overseas intensifies an enterprise’s institutional distance from its home country [21], reduces business information flow efficiency, and increases information asymmetry, leading to the liability of foreignness [22]. Stakeholders, including foreign governments, consumers, and investors [23], naturally pressurize MNEs because of their liability of origin. Despite the risks of disclosing information, including legal liability and exposure to the wrath of activists and stakeholders, recent studies have shown that international enterprises are willing to disclose environmental information to enhance their legitimacy [24, 25].

Unlike domestic enterprises, MNEs usually face institutional pressure in complex environments. For MNEs from emerging economies, their overseas production and operations face “disadvantage pressure” from the host country [26]. Therefore, their production and operations must exercise more caution, especially in terms of social responsibility and environmental protection. Given the improvements in their degree of internationalization, MNEs have become more dependent on overseas markets. The significance of environmental protection and disclosure requirements of stakeholders from host countries has increased, particularly that of image, legitimacy, and disclosure quality. A higher degree of internationalization means that more stakeholders are involved in supervising enterprise behavior [27], improving disclosure quality, promoting international cooperation, and instructing MNEs.

A study of UK firms using the FTSE 100 demonstrates that receiving environmental awards is positively related to environmental disclosure and helps MNEs enhance their legitimacy and brand reputation [24]. A higher level of internationalization involves more foreign stakeholders, reinforcing the risk of engaging in adverse situations [28] and offering enterprises more opportunities to access global norms and institutions [29]. Top-level MNEs excel in environmental disclosure, outperforming smaller MNEs and pursuing greater transparency.

Increasing environmental disclosure requires effort but is essential. When MNEs have higher levels of internationalization, they are more willing to enhance their legitimacy and reputation given the higher number of stakeholders, international norms, and legalization problems. Increasing environmental disclosure is a feasible, cost-effective method to enhance legitimacy and reputation in the host country. Thus, this study proposes the following hypothesis:

**Hypothesis 1:** Internationalization improves MNEs’ willingness to disclose environmental information.

Highly internationalized MNEs encounter more challenges in foreign markets [3] because of the differences in formal and informal institutions in foreign contexts [21] and their extensive presence in various countries or regions [28]. To achieve legitimacy in multiple countries, MNEs should consider the frequency and quality of environmental disclosure. Developed countries impose stricter legitimacy requirements than emerging economies [30]. Therefore, high-quality environmental disclosures can boost legitimacy in overseas markets by demonstrating a responsible corporate image [24]. Consequently, we posit the following hypothesis:

**Hypothesis 2:** Internationalization improves MNEs’ environmental disclosure quality.

### 2.3 Green investors

Previous studies on institutional investors have primarily focused on their impact on CSR [31–34]. Investors, driven by moral ethics, seek investments aligned with social goals, prioritizing values over personal gains [35]. Companies embracing sustainability and social responsibility address societal issues, bolstering their reputation and competitive edge, which benefits investors’ wealth [36]. Institutional investors belong to a particular group of financial institutions and include both green and non-green investors. The distinction lies in their primary goals of non-green investors prioritizing economic benefits and green investors focusing on green and high-quality investment [7].

Investors are essential stakeholders in enterprises and affect corporate governance. The traditional management concept often puts shareholders first, but according to the stakeholder theory [37], executives must balance all stakeholders’ rights and interests without focusing only on shareholders. Unlike traditional investors, green investors emphasize social responsibility investments [38] and have dual financial and environmental objectives [8, 9]. Accordingly, green investors that focus on environmental responsibility goals often achieve higher financial performance [39]. Enterprises with green investors are more likely to implement green actions, increase green spending, and improve their green governance performance. Further research shows that the promotional effect of green investors on green action is more evident in enterprises with weak environmental awareness. Specifically, green investors have promoted the reform of heavily polluting enterprises [40] by encouraging them to increase environmental investments, implement green actions, and improve environmental governance. Therefore, green actions and governance performance contribute to overall corporate performance.

Enterprises with green investors can signal their willingness to participate in green governance [41] and guarantee the smooth implementation of enterprises’ participation in green governance. This can prevent pollution penalties, help gain external recognition, and enhance green governance performance to some extent. Green investors aim for sustainable investments [42] and consider economic, social, environmental, and other factors. They encourage enterprises to pursue economic benefits while actively undertaking social responsibilities to obtain economic and social value [43]. Green investors encourage enterprises to participate in green governance, for which environmental disclosure is integral. Strong green governance in an enterprise fosters a positive public image and showcases a commitment to CSR. Unlike other stakeholders, investors control financial capital, which determines an enterprise’s development direction. Green investors prioritize environmental performance, and demand increased disclosure from enterprises to assess it. Thus, we propose the following hypothesis:

**Hypothesis 3**: Green investors strengthen the positive relationship between internationalization and environmental disclosure.

### 2.4 Heterogeneous effects

#### 2.4.1 MNEs with different ownership structures

SOEs are an organic part of national finance. Their social responsibility is an effective means for the state to participate in and intervene in the economy on behalf of the public interest [5]. SOEs’ social responsibility includes both economic and non-economic objectives. Social responsibility emphasizes non-economic goals, with economic goals helping to achieve non-economic goals. SOEs play an essential role in economic activities and may be the quickest government executors, corresponding to national industrial policies. Thus, SOEs contribute to national objectives [6].

Executives, especially those of SOEs, struggle to achieve the goals of “peak carbon dioxide emissions” and “carbon neutrality” proposed by governments worldwide [44]. Their remuneration and promotion as SOE executives primarily depend on successfully achieving these goals [45], of which environmental disclosure is an integral part. Therefore, SOE executives tend to be motivated to improve their environmental disclosure activities. Subject to governmental pressure, SOEs have more incentives to enhance their environmental disclosure willingness and quality than non-SOEs. Given that SOEs embody governmental authority, enhanced environmental disclosures can be strategic moves to secure legitimacy and reputation. Thus, we propose the following hypothesis:

**Hypothesis 4a**: Internationalization has a more pronounced positive effect on environmental disclosure for SOEs.

#### 2.4.2 Host countries with different development levels

MNEs operating in developed countries face more pressure compared to those in developing countries. The external pressure theory [2, 46] suggests that enterprises are compelled to disclose environmental information because of external pressure. The legitimacy requirement in developed countries is stricter than in developing countries. Stakeholders such as foreign governments, consumers, and investors [23] exert pressure on MNEs because of their origin. Moreover, stakeholders in developed countries have greater environmental awareness, and enterprises there possess better environmental management skills. Therefore, we propose the following hypothesis:

**Hypothesis 4b**: The positive effect of internationalization on environmental disclosure primarily occurs in developed host countries.

**Fig 1:**
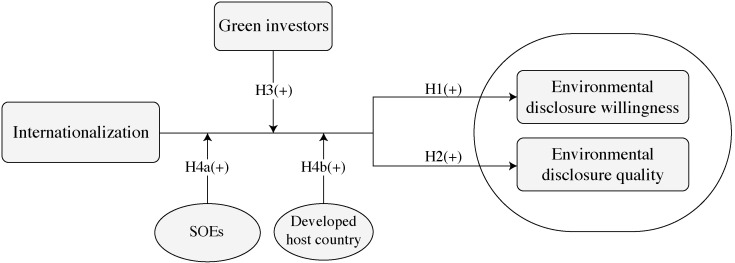
Logic graph.

The logic graph of this study is shown in Fig 1.

## 3. Variables, data, and methodology

### 3.1 Data

The sample includes publicly listed multinational companies on the Chinese domestic stock market from 2014 to 2021. Data on environmental disclosure, Chinese overseas subsidiaries, foreign sales, and green investors were obtained from China Stock Market and Accounting Research (CSMAR). The developed country list was obtained from the International Monetary Fund (IMF).

The following observations were excluded from the sample: (1) firms from “tax havens,” (2) Special Treatment (ST) firms, (3) financial enterprises, and (4) missing values and abnormal data. Additionally, all continuous variables were winsorized at the first and 99th percentiles. Next, we merged the data according to the year and firm stock codes. Finally, we obtained a dataset of 4,709 firm-year observations from 2014 to 2021.

### 3.2 Variables

#### 3.2.1 Dependent variables

*Willingness*. This was measured in three dimensions (disclosure of environmental management, disclosure of environmental supervision and certification, and environmental disclosure carrier) with 18 sub-indicators. Each sub-indicator was constructed as a dummy variable, taking a value of one if the enterprises had disclosed related information in their environmental report and zero otherwise [47]. Next, we constructed an indicator to measure willingness for environmental disclosure by summing all 18 sub-indicators. The final total score (hundred-mark) was used to measure the willingness to engage in environmental disclosure, namely “Willingness = number of disclosure items/18*100.” S1 Appendix provides detailed information on sub-indicators.

*Quality*. We used the disclosure index of environmental information (EID), which is often used in environmental disclosure research [47]. We used two dimensions (environmental liability and environmental disclosure performance and governance) with 12 sub-indicators using qualitative or quantitative data. The score of the sub-indicator was zero if the enterprise did not disclose related information, one if the enterprise only qualitatively disclosed, and two if the enterprise quantitatively disclosed. Thus, if an enterprise quantitatively disclosed all 12 sub-indicators, its total score was 24. Subsequently, we added all 12 sub-indicators and the final total score (hundred mark) was used to measure the quality of environmental disclosure, namely “Quality = disclosure score/24*100.” S1 Appendix provides information on sub-indicators.

#### 3.2.2 Independent variable

*Internationalization (FSALE)*. We employed the ratio of overseas sales to total sales to measure internationalization level [47].

#### 3.2.3 Moderating variables

*Green investors (GI)*. The original data were obtained from the CSMAR. We matched fund information data with stock investment data to obtain detailed data on funds in listed enterprises. Next, we used the content analysis method to verify whether the investment goal and investment scope contained environment-related keywords, including “environmental protection,” “ecological,” “green,” “new energy development,” “clean energy,” “low carbon,” “sustainable,” and “energy saving.” If the investment goal and scope contained these keywords, we considered them green investors [48]. Finally, we calculated enterprises’ number of green investors as GI = ln (number of green investors + 1).

#### 3.2.4 Control variables

We used *firm size (Size)*, *profitability (ROA)*, *market value (TobinQ)*, *CEO duality (Dual)*, *board size (Board)*, *and board independence (Indep)* as control variables. Firm size is widely used in the literature on firms’ environmental behavior [47]. We measured firm size using the natural logarithm of fixed assets. Higher profitability implies more resources to support better corporate environmental performance. We used return on assets (ROA) to measure it [49]. Market value is an investor’s assessment of the value of an enterprise and an aspect of corporate financial performance. We used Tobin’s Q to measure an enterprise’s market value [50]. CEO duality is a dummy variable that takes a value of one when the CEO is also the chairman of the board, and zero otherwise. Some studies show that the CEO also being the chairman of the board decreases benefits to shareholders, supervision power, and corporate information transparency [19]. We measured the board size based on the number of directors. Board size influences corporate governance. A large board suffers from poor coordination, increases the complexity of decision-making [51], and may influence an enterprise’s environmental disclosure decisions. We defined board = ln (number of directors + 1). We measured board independence using the ratio of independent directors to the entire board. High board independence minimizes agency problems and limits managerial problems [52, 53].

#### 3.2.5 Summary statistics

Table 1 presents descriptive statistics for the variables used in our regression analysis. Table 2 presents the correlation matrices for these variables. The correlations between variables are relatively low, indicating no major multicollinearity problems.

**Table 1:** Descriptive statistics.

Variable	Obs.	Mean	Std. dev.	Min	Max
Willingness	4709	19.034	15.297	0	83.333
Quality	4709	11.633	16.862	0	79.167
FSALE	4709	0.230	0.221	0	0.964
GI	4709	0.661	0.814	0	4.277
Size	4709	22.214	1.167	19.716	26.430
ROA	4709	0.036	0.080	-0.398	0.254
TobinQ	4709	2.109	1.294	0.802	17.729
Dual	4709	0.377	0.485	0	1.000
Board	4709	2.078	0.204	1.609	2.708
Indep	4709	0.383	0.056	0.286	0.600

**Table 2:** Correlation matrix.

Variables	(1)	(2)	(3)	(4)	(5)	(6)	(7)	(8)	(9)	(10)
**(1) Willingness**	1.000									
**(2) Quality**	0.755	1.000								
**(3) FSALE**	0.091	0.109	1.000							
**(4) GI**	0.166	0.149	-0.069	1.000						
**(5) Size**	0.476	0.425	-0.170	0.344	1.000					
**(6) ROA**	0.071	0.071	0.036	0.266	-0.021	1.000				
**(7) TobinQ**	-0.185	-0.144	0.088	0.181	-0.359	0.182	1.000			
**(8) Dual**	-0.114	-0.106	0.070	0.016	-0.168	0.021	0.084	1.000		
**(9) Board**	0.179	0.170	-0.080	0.050	0.234	0.024	-0.115	-0.130	1.000	
**(10) Indep**	-0.016	-0.007	0.026	0.024	0.004	-0.009	0.036	0.104	-0.586	1.000

### 3.3 Methods

Considering that the data have a panel structure and that the dependent variables (willingness and quality) are continuous, we used a panel data-related model. First, we used the Hausman test to analyze whether the fixed or random effects models were suitable [54]. The test results support the use of the fixed effect model, which is a typical method for panel data analysis. To minimize the impact of missing variable bias, we selected a dual fixed effect model to analyze the data, control firm, and year. The models are shown in Eqs (1) and (2):

$${Willingness}_{it}=\alpha_{0}+\beta_{1}{FSALE}_{it}+\beta_{2}{GI}_{it}+\beta_{3}{{FSALE}_{it}\times GI}_{it}+\lambda X_{it}+\gamma_{i}+\omega_{t}+\varepsilon_{it}$$ (1)

$${Quality}_{it}=\alpha_{0}+\beta_{1}{FSALE}_{it}+\beta_{2}{GI}_{it}+\beta_{3}{{FSALE}_{it}\times GI}_{it}+\lambda X_{it}+\gamma_{i}+\omega_{t}+\varepsilon_{it}$$ (2)

Subscripts *i* and *t* denote firm and year, respectively. *Willingness* is environmental disclosure willingness and *Quality* is environmental disclosure quality. *X* is a vector representing the control variables. Firm (*γ*_*i*_) and year (*ω*_*t*_) fixed effects are included in the model.

## 4. Empirical analysis

### 4.1 Overall and multi-dimensional regression

Table 3 presents results for the effect of internationalization on environmental disclosure willingness. Column (1) displays the results for the model with the key independent variable. Column (2) displays results of introducing control variables. The coefficients of *FSALE* are significantly positive shown in Column (2), verifying that internationalization positively affects environmental disclosure willingness. To deepen our analysis, we divide environmental disclosure willingness into the following three dimensions: (a) environmental management, (b) environmental supervision and certification, and (c) environmental disclosure carriers. The regression results for the three dimensions are also listed in Table 3. The coefficients of *FSALE* in Columns (3) and (4) are significantly positive at 5% and 1% levels. Only the coefficient of *FSALE* on supervision and certification dimension is insignificant as shown in Column (5).

**Table 3:** The effect of internationalization on environmental disclosure willingness (Overall and detailed dimensional regressions).

	(1)	(2)	(3)	(4)	(5)
Variables	Willingness	Willingness	Environmental management	Disclosure carrier	Supervision and certification
**FSALE**	3.039	3.842*	0.463**	0.219***	0.0932
	(2.071)	(2.041)	(0.220)	(0.0832)	(0.159)
**Size**		3.340***	0.403***	0.105***	0.0684
		(0.657)	(0.0723)	(0.0280)	(0.0483)
**ROA**		2.012	0.225	-0.239***	0.236
		(1.994)	(0.211)	(0.0756)	(0.156)
**Dual**		-0.383	-0.0400	0.00924	-0.0169
		(0.507)	(0.0573)	(0.0218)	(0.0379)
**TobinQ**		0.742***	0.0880***	0.00668	0.0266*
		(0.187)	(0.0209)	(0.00941)	(0.0142)
**Board**		-1.219	-0.140	0.0143	-0.0669
		(2.002)	(0.240)	(0.0818)	(0.153)
**Indep**		4.116	0.491	0.121	0.216
		(5.552)	(0.635)	(0.240)	(0.464)
**Firm FE**	Yes	Yes	Yes	Yes	Yes
**Year FE**	Yes	Yes	Yes	Yes	Yes
**Constant**	14.43***	-58.51***	-7.695***	-1.470**	0.0217
	(1.270)	(15.64)	(1.735)	(0.663)	(1.123)
**Observations**	4,709	4,709	4,709	4,709	4,704
**R-squared**	0.210	0.225	0.129	0.168	0.079

Note: Robust standard errors are between parentheses

*** p<0.01

** p<0.05

* p<0.1

A higher level of internationalization requires deeper integration into the host country and generates stronger incentives for MNEs to reinforce environmental disclosure. External pressure [46] from the host country pushes MNEs to improve environmental disclosure to gain legitimacy, reduce information asymmetry, and overcome the liability of foreignness [2]. Therefore, Hypothesis 1 is verified.

Table 4 presents the results for the effect of internationalization on environmental disclosure quality. Column (1) displays the results of the model with the key independent variable. Column (2) displays the results of introducing control variables. The coefficients of *FSALE* are significantly positive, as shown in Columns (1) and (2), verifying that internationalization positively affects environmental disclosure quality. We also examine the relationship between internationalization and the two detailed dimensions (disclosure of environmental liability and disclosure of environmental performance and governance) of environmental disclosure quality. Table 4 presents the results. The coefficient of *FSALE* is significantly positive at the 5% level for the performance and governance dimension, as shown in Column (4).

**Table 4:** The effect of internationalization on environmental disclosure quality (Overall and detailed dimensional regressions).

	(1)	(2)	(3)	(4)
Variables	Quality	Quality	Environmental liability	Performance and governance
**FSALE**	3.793*	4.441*	2.830	6.328**
	(2.241)	(2.287)	(2.451)	(2.650)
**Size**		2.730***	2.226**	3.304***
		(0.893)	(1.041)	(0.910)
**ROA**		4.866**	5.074**	4.602*
		(2.062)	(2.170)	(2.497)
**Dual**		-0.113	-0.403	0.180
		(0.675)	(0.714)	(0.807)
**TobinQ**		0.678***	0.693***	0.682***
		(0.189)	(0.205)	(0.219)
**Board**		-1.460	-4.594*	1.125
		(2.149)	(2.439)	(2.521)
**Indep**		2.010	-7.752	10.70
		(6.858)	(7.482)	(7.899)
**Firm FE**	Yes	Yes	Yes	Yes
**Year FE**	Yes	Yes	Yes	Yes
**Constant**	6.167***	-52.39**	-32.72	-72.61***
	(1.486)	(20.93)	(24.39)	(21.55)
**Observations**	4,709	4,709	4,709	4,709
**R-squared**	0.126	0.137	0.121	0.103

Note: Robust standard errors are between parentheses

*** p<0.01

** p<0.05

* p<0.1

The environmental disclosure by enterprises primarily results from external pressure [2, 46], which can be divided into the following two categories: pressure from the government for legitimacy and pressure from the public. The former is exerted through the implementation of laws and regulations, representing direct pressure. The latter manifests as public opinion and market behavior, representing indirect pressure. According to the interpretation of external pressure theory [46], external stakeholders care about the environmental behavior of MNEs and prioritize the quality of this behavior, supporting Hypothesis 2.

### 4.2 Moderating effect of green investors

Table 5 presents results for the moderating effect of green investors on the relationship between internationalization and environmental disclosure willingness and quality. The coefficients of the interaction term between green investors and internationalization are significantly positive at the 1% level for the overall effect, as shown in Columns (1) and (5); this demonstrates that green investors strengthen the positive effect of internationalization on environmental disclosure willingness and quality.

**Table 5:** Moderating effect of green investors on environmental disclosure willingness and quality.

	(1)	(2)	(3)	(4)	(5)	(6)	(7)
Variables	Willingness	Environmental management	Disclosure carrier	Supervision and certification	Quality	Environmental liability	Performance and governance
FSALE	5.048**	0.544**	0.224**	0.192	6.321***	4.617*	8.380***
	(2.108)	(0.232)	(0.0879)	(0.163)	(2.385)	(2.595)	(2.743)
GI	1.048***	0.112***	-0.00759	0.0575***	1.014***	1.077***	1.022**
	(0.298)	(0.0333)	(0.0126)	(0.0220)	(0.372)	(0.416)	(0.417)
FSALE×GI	3.715***	0.227	0.0216	0.319***	6.158***	5.784***	6.771***
	(1.322)	(0.155)	(0.0558)	(0.0957)	(1.632)	(1.882)	(1.826)
Control variables	Yes	Yes	Yes	Yes	Yes	Yes	Yes
Firm FE	Yes	Yes	Yes	Yes	Yes	Yes	Yes
Year FE	Yes	Yes	Yes	Yes	Yes	Yes	Yes
Constant	-49.05***	-6.674***	-1.541**	0.535	-43.35**	-23.08	-63.52***
	(15.57)	(1.721)	(0.665)	(1.121)	(20.31)	(23.31)	(21.48)
Observations	4,709	4,709	4,709	4,704	4,709	4,709	4,709
R-squared	0.230	0.133	0.168	0.084	0.144	0.126	0.108

Note: Robust standard errors are between parentheses

*** p<0.01

** p<0.05

* p<0.1

According to stakeholder theory [37], executives of enterprises must balance the interests of all stakeholders, not just shareholders. Investors are important stakeholders of enterprises. Compared with traditional investors, green investors focus more on green and high-quality investment [7]. Green investors assign importance to green development [8], which exerts pressure on enterprises to improve environmental disclosure performance. Green investors have both financial and environmental objectives [8, 9]. Enterprises with green investors are more likely to take green actions. The mechanism of the positive moderating effect of green investors has two aspects. First, investors are important stakeholders of enterprises and greatly influence the overseas development strategy of MNEs. Green investors focus on green and high-quality development [7], pushing MNEs to focus on environmental performance in overseas markets. Environmental disclosure is an important aspect of environmental performance. Thus, green investors push MNEs to perform well on environmental disclosure. Second, green investors can relieve the financial constraints [55] and require MNEs to increase environmental expenditure. This is beneficial for MNEs to improve environmental performance. When a MNE has a better environmental performance, it is more willing to disclosure environmental information [12]. Therefore, green investors enhance the positive effect of internationalization on environmental disclosure, supporting Hypothesis 3.

**Fig 2:**
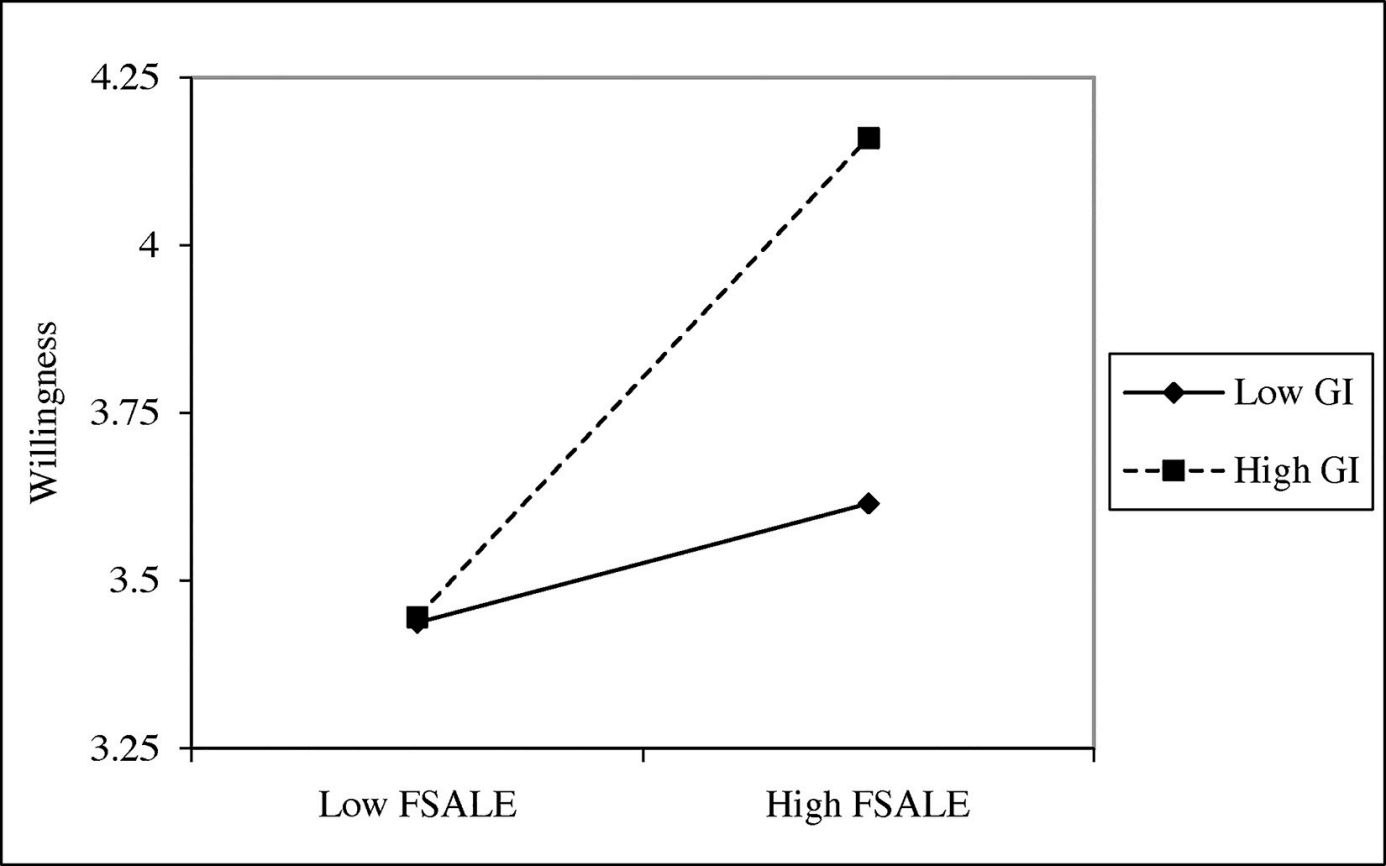
Moderating effect of green investors on environmental disclosure willingness.

**Fig 3:**
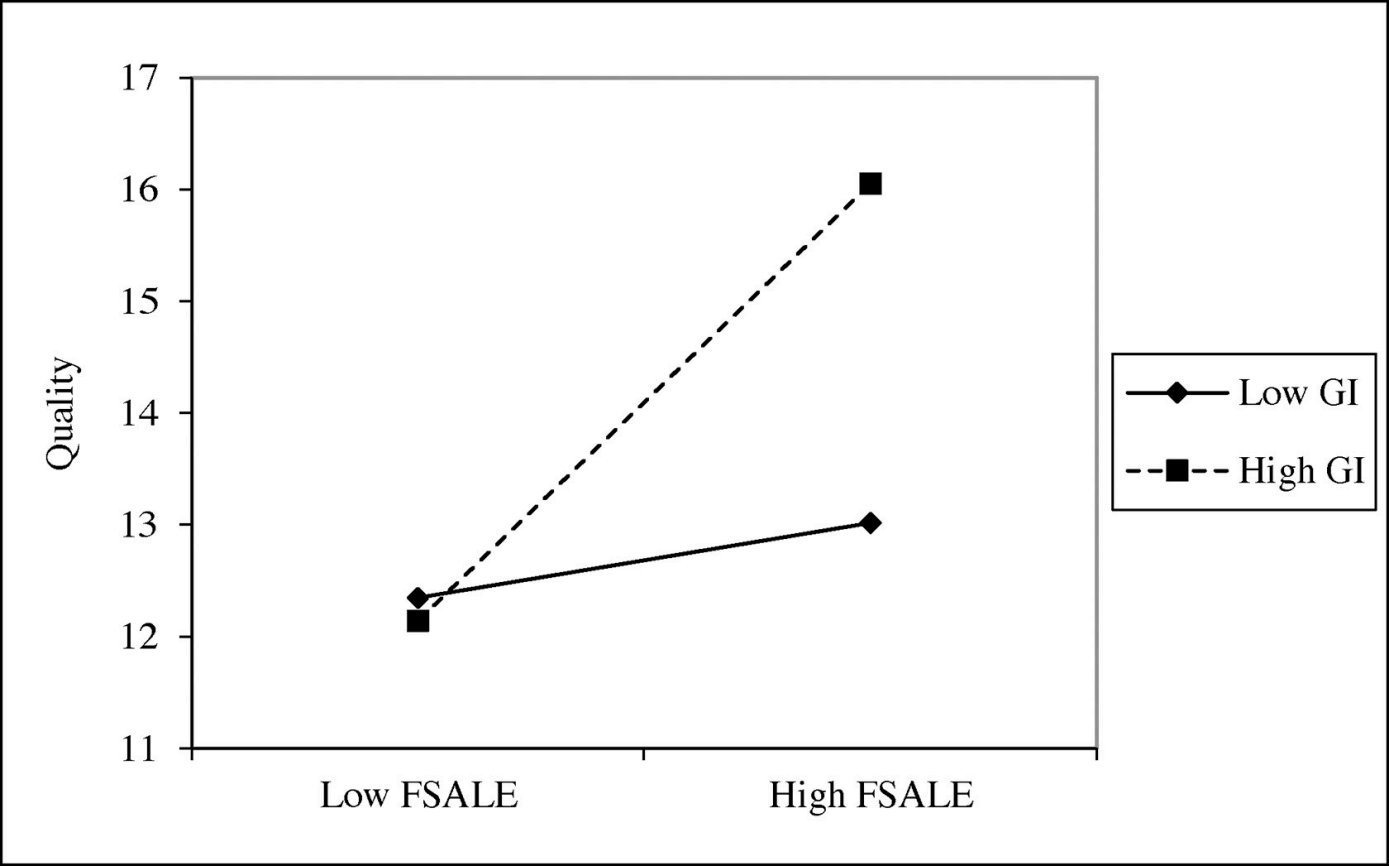
Moderating effect of green investors on environmental disclosure quality.

We demonstrate the moderating effect using the values one standard deviation above and below the mean to represent high and low levels of the moderating variables [3, 56]. The moderating effect of green investors on the relationship between internationalization and environmental disclosure willingness is shown in Fig 2. The moderating effect of green investors on the relationship between internationalization and environmental disclosure quality is shown in Fig 3.

Fig 2 shows that the slope becomes steeper when both *Willingness* and *FSALE* are high. This indicates that as *FSALE* increases from one standard deviation below the mean to one above, *Willingness* shows a faster increase when *GI* is higher. In Fig 3, the slope is also steeper when both *Quality* and *FSALE* are high. This indicates that as *FSALE* increases from one standard deviation below the mean to one above, *Quality* demonstrates a faster increase when *GI* is higher.

### 4.3 Heterogeneity analysis

#### 4.3.1 SOEs and non-SOEs

We construct a dummy variable (SOE) to divide the MNEs into state-owned and non-state-owned. The dummy variable takes the value of one if the enterprise is state-owned; otherwise, it takes zero. Table 6 shows the results. The coefficients of *FSALE* and the interaction term are significantly positive at the 1% level. Next, we use the Chow test to verify whether there is a coefficient difference between groups. The result indicates that there is difference between groups. The positive effect of internationalization on environmental disclosure is stronger for SOEs (Columns (1) and (2): *β* = 15.19>9.142, p < .01; Columns (3) and (4): *β* = 21.33>11.26, p < .01).

**Table 6:** SOEs and non-SOEs.

	(1)	(2)	(3)	(4)
Variables	Willingness (SOEs)	Willingness (Non-SOEs)	Quality (SOEs)	Quality (Non-SOEs)
FSALE	15.19***	9.142***	21.33***	11.26***
	(4.046)	(1.387)	(4.346)	(1.517)
GI	1.641**	1.012***	1.428	1.115***
	(0.738)	(0.285)	(0.971)	(0.339)
FSALE×GI	6.509**	6.902***	14.83***	10.24***
	(2.996)	(1.269)	(3.994)	(1.446)
Control variables	Yes	Yes	Yes	Yes
Fixed effect	Yes	Yes	Yes	Yes
Constant	-146.0***	-100.4***	-179.7***	-101.5***
	(19.97)	(10.21)	(22.61)	(11.68)
Observations	810	3,899	810	3,899

Note: Robust standard errors are between parentheses

*** p<0.01

** p<0.05

* p<0.1

SOEs aim to achieve economic and national goals [5, 6], and green development is currently a global and national objective. Executives, especially those of SOEs, face challenges in meeting the “peak carbon dioxide emissions” and “carbon neutrality” goals set by governments worldwide [44]. Furthermore, the remuneration and promotion of SOE executives largely rely on successfully achieving these goals [45]. Therefore, SOEs tend to enhance their environmental disclosure activities, supporting Hypothesis 4a.

#### 4.3.2 Developed and developing host countries

We categorize the host countries as either developed or developing countries. Table 7 shows the empirical results. The impact of internationalization on both the willingness and quality of environmental disclosure, as well as the moderating effect of green investors, is significant in developed host countries (Column (1) and Column (3)). Conversely, the results are insignificant in developing host countries (Column (2) and Column (4)).

**Table 7:** Developed and developing host countries.

	(1)	(2)	(3)	(4)
Variables	Willingness (Developed host countries)	Willingness (Developing host countries)	Quality (Developed host countries)	Quality (Developing host countries)
**FSALE**	9.217***	2.778	12.13***	10.42
	(2.459)	(5.222)	(2.932)	(6.570)
**GI**	0.310	1.162	0.375	1.013
	(0.767)	(1.632)	(0.668)	(1.585)
**FSALE×GI**	5.148**	4.562	12.20***	4.356
	(2.526)	(4.695)	(2.288)	(6.424)
**Control variables**	Yes	Yes	Yes	Yes
**Fixed effect**	Yes	Yes	Yes	Yes
**Constant**	-141.4***	-95.1**	-157.1***	-112.1***
	(16.70)	(37.41)	(18.25)	(37.24)
**Observations**	1,722	349	1,722	349

Note: Robust standard errors are between parentheses

*** p<0.01

** p<0.05

* p<0.1

Developed countries have more stringent legitimacy requirements compared to developing countries. Stakeholders, such as foreign governments, consumers, and investors [23], naturally exert pressure on MNEs because of their liability of origin. The environmental awareness of stakeholders from developed countries also surpasses that of developing ones. Consequently, the positive effect of internationalization on environmental disclosure, along with the moderating effect of green investors, is primarily associated with developed host countries. Thus, Hypothesis 4b is supported.

### 4.4 Endogeneity

One primary concern is the potential endogeneity due to reverse causality and omitted variable bias. To address the potential endogeneity issue, we introduce an instrumental variable. Following literature addressing endogeneity in similar situations, the industrial average of internationalization level is utilized as the instrumental variable [57, 58]. If the endogeneity is firm-specific but not industry-specific, netting out this firm-specific component yields a measure that depends only on the underlying characteristics of a particular industry [57]. The results are shown in Table 8. In the first stage, we find that the instrumental variable is effective, as demonstrated by the F-value of 149.99 (which exceeds the threshold of 10). In the second stage, the regression results are consistent with the baseline findings.

**Table 8:** Instrumental variable method.

	1st stage	2nd stage	
	(1)	(2)	(3)
**VARIABLES**	**FSALE**	**Willingness**	**Quality**
**IV (FSALEave)**	0.342***		
	(0.0280)		
**FSALE**		22.31***	25.15***
		(2.151)	(2.352)
**GI**		1.063***	1.027***
		(0.318)	(0.354)
**FSALE×GI**		10.69***	14.18***
		(2.754)	(3.079)
**Control variables**	Yes	Yes	Yes
**Fixed effect**	Yes	Yes	Yes
**Constant**	0.731***	-144.6***	-158.3***
	(0.104)	(6.025)	(6.881)
**F-statistic**	149.99***		
**Observations**	4,709	4,709	4,709
**R-squared**	0.058	0.311	0.272

Note: Robust standard errors are between parentheses

*** p<0.01

** p<0.05

* p<0.1

### 4.5 Robustness test

To enhance the reliability of the empirical findings, we perform a robustness test. This includes employing a lagged structure for all independent variables, a Tobit model, and an alternative independent variable to ensure the consistency of the results. Table 9 shows the robustness test results.

**Table 9:** Robustness test.

	Lagged independent variables		Tobit model		Alternative independent variable	
	(1)	(2)	(3)	(4)	(5)	(6)
VARIABLES	Willingness	Quality	Willingness	Quality	Willingness	Quality
FSALE			10.40***	20.90***		
			(1.151)	(2.571)		
GI			0.912***	0.178	0.843***	0.681*
			(0.245)	(0.539)	(0.288)	(0.348)
FSALE×GI			6.467***	15.89***		
			(1.074)	(2.298)		
l.FSALE	14.46***	17.94***				
	(1.118)	(1.250)				
l.GI	0.888**	0.938**				
	(0.353)	(0.413)				
l.FSALE×GI	11.47***	17.77***				
	14.46***	17.94***				
FCNT					2.115**	3.688***
					(0.837)	(0.951)
FCNT×GI					1.307**	1.788***
					(0.589)	(0.681)
Control variables	Yes	Yes	Yes	Yes	Yes	Yes
Fixed effect	Yes	Yes	No	No	Yes	Yes
Constant	-135.0***	-147.7***	-115.1***	-234.7***	-41.12***	-31.65
	(6.885)	(8.013)	(6.806)	(15.13)	(15.56)	(20.30)
R-squared	0.277	0.238			0.230	0.146
Observations	3,195	3,195	4,709	4,709	4,709	4,709

Note: Robust standard errors are between parentheses

*** p<0.01

** p<0.05

* p<0.1

#### 4.5.1 Lagged structure for independent variables

One primary concern is that *FSALE* may be endogenous because of a reverse causality problem, meaning that firms with high environmental disclosure performance have a high degree of internationalization. The problem of two-way causality can be weakened by using lagged independent variables [4, 57]. The current dependent variable does not influence the lagged independent variable. The empirical results are shown in Table 9. The coefficients of the lagged key independent variable *l*.*FSALE* and the interaction term are significantly positive at the 1% level, as shown in Columns (1) and (2). The results are consistent with those of the baseline model.

#### 4.5.2 Replacement with a new estimation method

The dependent variable (*willingness*, *quality*) is an enterprise’s environmental disclosure willingness or quality score; if an enterprise does not disclose any environmental item, the score is zero A silent non-disclosure enterprise is also an option in a partial disclosure equilibrium setting [59]. Hence, the Tobit model is suitable [60], and we use it for re-estimation; its general form is given in the following Eq (3):

$$y_{it}^{*}=X_{it}^{\prime }\beta +\varepsilon_{it}$$ (3)

$$y_{it}=y_{it}^{*}\;y_{it}^{*}>0$$

$$y_{it}=0\;y_{it}^{*}\leq 0$$

Here, $y_{it}^{*}$ is the unobserved variable, *y*_*it*_ is the observed variable, $X_{it}^{\prime }$ is the explanatory variable vector, and *ε*_*it*_ is the random disturbance term. The empirical results are shown in Table 9. The coefficients of the key independent variable *FSALE* and the interaction term are significantly positive at the 1% level, as shown in Columns (3) and (4). The results are consistent with those of the baseline model.

#### 4.5.3 Alternative independent variable

To ensure the reliability of our results, we substitute the independent variable *FSALE* with *FCNT* (number of foreign countries that MNEs invest in overseas markets) [61], defining *FCNT* = ln (number of foreign countries investing overseas + 1). Table 9 presents the empirical results. The coefficients of the key independent variable *FCNT* and the interaction term are significantly positive at the 5% and 1% levels, as shown in Columns (5) and (6). The results are consistent with those of the baseline model.

## 5. Conclusion and discussion

### 5.1 Conclusion

This study concludes that a higher level of internationalization can improve MNEs’ willingness and quality of environmental disclosure, and green investors strengthen this positive relationship. Heterogeneity analysis shows that the positive relationship is primarily associated with SOEs and developed host countries. Higher level of internationalization requires deeper integration into the host country and generates stronger incentive for MNEs to reinforce environmental disclosure. External pressure [46] from the host country pushes MNEs to improve environmental disclosure for gaining legitimacy, reducing information asymmetry and overcoming the liability of foreignness [2]. Green investors are important stakeholders of enterprises. Compared to traditional investors, green investors focus more on green and high-quality investment [7], contributing to relieving financial constraints of MNEs [55] and pushing them to improve environmental disclosure performance.

### 5.2 Implications

Our study contributes to the literature in several aspects. First, it introduces “environmental disclosure willingness” and “environmental disclosure quality”, refining traditional environmental disclosure research [12–15, 18]. Second, our study explores environmental disclosure from an international perspective, expanding on the existing domestic perspective [19, 41]. Third, considering the increasing attention to environmental issues, our study focuses on the important role of green investors, deepening traditional investor research [9, 38].

Our study offers some recommendations for MNEs, especially those from emerging economies. First, it points to the importance of utilizing environmental disclosure to achieve legitimacy in international contexts. Executives should attach importance to environmental disclosure when MNEs enter overseas markets. Second, it proposes the importance of green investors given the increasing attention to environmental protection. MNEs are recommended to introduce green investors for improving environmental performance. Third, it provides theoretical insights for environmental policy reform. With the implementation of green finance, green investors are playing a more important role in environmental disclosure. Policy makers should formulate more specific policies that encourage enterprises to welcome green investors, who guide corporate environmental behavior as emerging strategic investors.

### 5.3 Limitations and scope for future research

While we have examined the link between internationalization and environmental disclosure, our analysis is subject to some limitations. First, we subdivide environmental disclosure willingness into three dimensions and environmental disclosure quality into two dimensions. While the overall empirical result and moderating effects are significant, not all detailed dimensions show significant results. Further research is needed to find out the underlying reason. Second, our study considers only the moderating role of green investors. Stakeholders play an important role in corporate environmental management. Further research should introduce other stakeholders to enrich the research results.

## Supporting information

S1 Data Regression data. (XLS)

S1 Appendix Environmental disclosure items. (DOCX)

S1 Graphical abstract (TIF)
