# Supplementary material for: Does internationalization improve environmental disclosure willingness and quality? The moderating role of green investors
Source: PLoS One. 2024 Sep 11;19(9):e0307638. doi: 10.1371/journal.pone.0307638 (PMC11389905; doi:10.1371/journal.pone.0307638)
Supplement: S1 Appendix — (DOCX) [file pone.0307638.s002.docx]

**S1 Appendix. Environmental disclosure items.**

| **Environmental disclosure** | **Classification** | **Environmental disclosure item** |
| --- | --- | --- |
| Environmental disclosure willingness | Environmental Management | (1) Environmental Protection Concept |
|  |  | (2) Environmental Protection Goal |
|  |  | (3) Environmental Management System Schema |
|  |  | (4) Environmental Education and Training |
|  |  | (5) Environmental Protection Special Act |
|  |  | (6) Environmental Event Emergency Mechanism |
|  |  | (7) Environmental Protection Honor Reward |
|  |  | (8) Three Simultaneity System |
|  | Environmental Supervision and Certification | (9) Key Pollution Monitoring Unit |
|  |  | (10) Pollution Emission Standard |
|  |  | (11) Sudden Environmental Accident |
|  |  | (12) Environmental Violation |
|  |  | (13) Environmental Petition Letter Case |
|  |  | (14) Is Pass ISO14001 |
|  |  | (15) Is Pass ISO9001 |
|  | Environmental Disclosure Carrier | (16) Annual Report |
|  |  | (17) CSR Report |
|  |  | (18) Environment Report |
| Environmental disclosure quality | Environmental Disclosure Performance and Governance | (1) Waste Gas Emission Reduction |
|  |  | (2) Wastewater Emission Reduction |
|  |  | (3) Soot Dust Reduction |
|  |  | (4) Solid Waste Disposal Utilization |
|  |  | (5) Noise Light Radiation Governance |
|  |  | (6) Clear Production Implementation |
|  | Environmental Liability | (7) Wastewater Emissions |
|  |  | (8) COD Emissions |
|  |  | (9) SO_2_ Emissions |
|  |  | (10) CO_2_ Emissions |
|  |  | (11) Soot Dust Emissions |
|  |  | (12) Industry Solid Waste Emissions |
