# Supplementary figures and images for: Does internationalization improve environmental disclosure willingness and quality? The moderating role of green investors
Source: PLoS One. 2024 Sep 11;19(9):e0307638. doi: 10.1371/journal.pone.0307638 (PMC11389905; doi:10.1371/journal.pone.0307638)

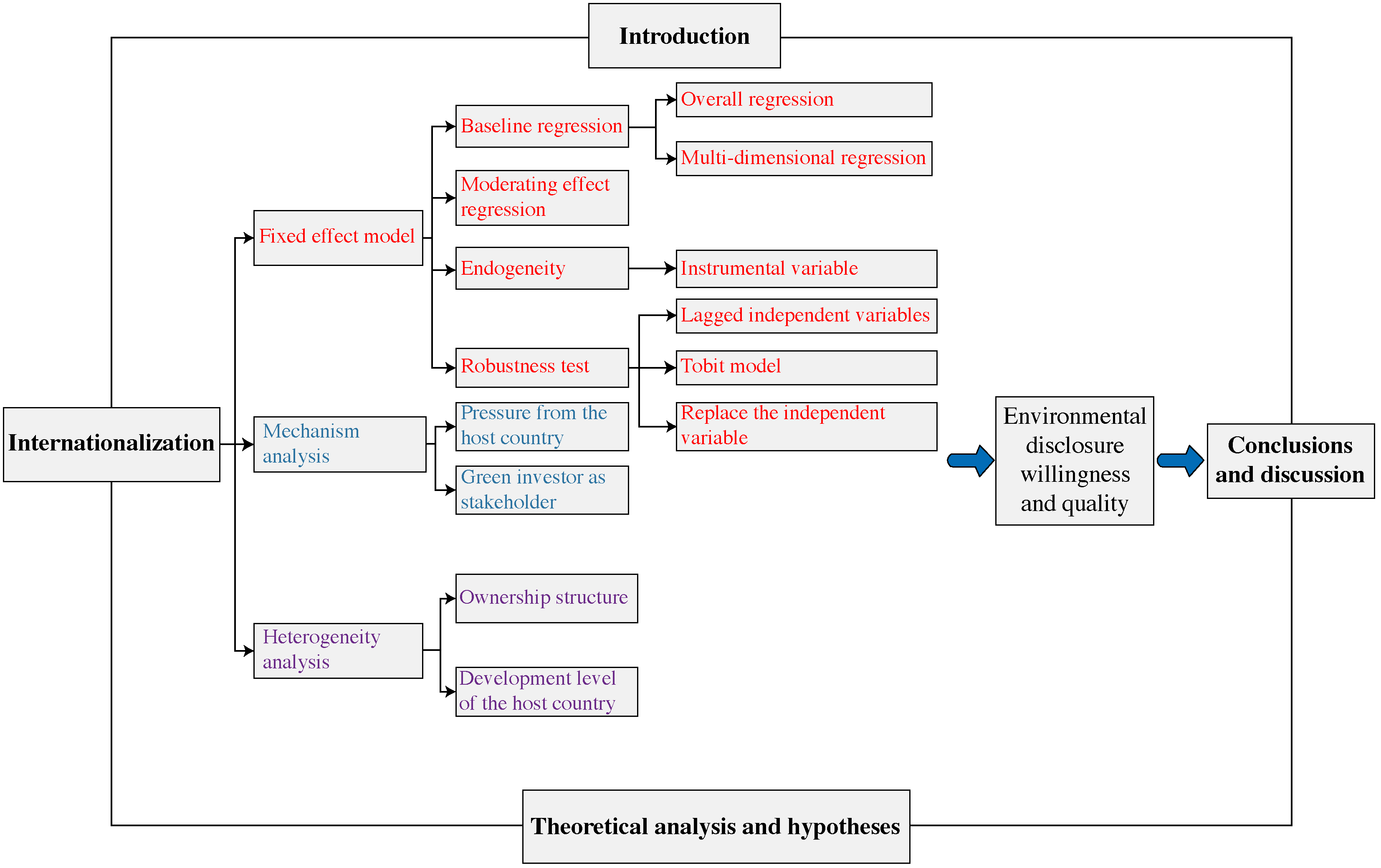

Supplement: S1 Graphical abstract — (TIF) [file pone.0307638.s003.tif]
